# G Protein-Coupled Estrogen Receptor Correlates With Dkk2 Expression and Has Prognostic Impact in Ovarian Cancer Patients

**DOI:** 10.3389/fendo.2021.564002

**Published:** 2021-02-19

**Authors:** Patricia Fraungruber, Till Kaltofen, Sabine Heublein, Christina Kuhn, Doris Mayr, Alexander Burges, Sven Mahner, Philipp Rathert, Udo Jeschke, Fabian Trillsch

**Affiliations:** ^1^ Department of Obstetrics and Gynecology, University Hospital, Ludwig-Maximilians-University (LMU) Munich, Munich, Germany; ^2^ Department of Gynecology and Obstetrics, University of Heidelberg, Heidelberg, Germany; ^3^ Department of Pathology, LMU Munich, Munich, Germany; ^4^ Department of Biochemistry, University Stuttgart, Stuttgart, Germany; ^5^ Department of Obstetrics and Gynecology, University Hospital Augsburg, Augsburg, Germany

**Keywords:** Dickkopf 2, G protein-coupled estrogen receptor, Wnt signaling, estrogen, epithelial ovarian cancer

## Abstract

**Purpose:**

Wnt pathway modulator Dickkopf 2 (Dkk2) and signaling of the G protein-coupled estrogen receptor (GPER) seem to have essential functions in numerous cancer types. For epithelial ovarian cancer (EOC), it has not been proven if either Dkk2 or the GPER on its own have an independent impact on overall survival (OS). So far, the correlation of both factors and their clinical significance has not systematically been investigated before.

**Methods:**

Expression levels of Dkk2 were immunohistochemically analyzed in 156 patient samples from different histologic subtypes of EOC applying the immune-reactivity score (IRS). Expression analyses were correlated with clinical and pathological parameters to assess for prognostic relevance. Data analysis was performed using Spearman’s correlations, Kruskal-Wallis-test and Kaplan-Meier estimates.

**Results:**

Highest Dkk2 expression of all subtypes was observed in clear cell carcinoma. In addition, Dkk2 expression differed significantly (p<0.001) between low and high grade serous ovarian cancer. A significant correlation of Dkk2 with the cytoplasmic GPER expression was noted (p=0.001) but not for the nuclear estrogen receptor alpha (ERα) or beta (ERβ). Patients exhibiting both, high expression Dkk2 (IRS>4) and GPER (IRS>8), had a significantly better overall survival compared to patients with low expression (61 months vs. 33 months; p=0.024).

**Conclusion:**

Dkk2 and GPER expression correlates in EOC and combined expression of both is associated with improved OS. These findings underline the clinical significance of both pathways and indicate a possible prognostic impact as well as a potential for treatment strategies addressing interactions between estrogen and Wnt signaling in ovarian cancer.

## Introduction

Epithelial ovarian cancer (EOC) causes most deaths of gynecological malignancies (1) with a relative 5-year survival of almost 45% (2). The need to identify suitable screening methods, prognostic markers and efficient therapies is crucial. So far, standard treatment for primary disease consists of debulking surgery and a platinum-based chemotherapy with antiangiogenics and/or Poly-ADP-Ribose-Polymerase (PARP) inhibitors (3). Apart from clinicopathological aspects such as the stage in the system of the International Federation of Gynecology and Obstetrics (FIGO), volume of residual disease after debulking surgery, patients’ age, and histological subtype (4–7), there are no reliable prognostic factors to predict the clinical course. With regards to the molecular background and specific gene mutations, EOC is histologically separated into clear cell, endometrioid, mucinous, and serous carcinoma of low or high grade (LGSC/HGSC) (8).

Revealing molecular events that cause ovarian cancer and are responsible for its progression represent a major challenge for translational research. One approach is to understand the importance and complexity of the Wnt signaling pathway and its regulation (9–11). Secreted Wnt glycoproteins translate their function *via* binding to Frizzled receptors and co-receptors such as low-density-lipoprotein-related protein 5/6 (LRP5/6) (11). Subsequently, Wnt proteins exhibit their effects on several cellular processes by activating either the canonical Wnt/β-catenin or at least two non-canonical β-catenin-independent pathways (12). Alterations in Wnt signaling components, such as APC (adenomatous polyposis coli) protein, AXIN and β-catenin and downregulation of modulatory Wnt antagonists have been described to be involved in the onset of several cancer types (10, 13, 14). As a consequence, modulators of the Wnt pathway like members of the Dickkopf family (Dkk1-4) may play an essential role during development (15, 16) and tumorigenesis (17, 18). Dkks bind to LRP5/6 with higher affinity than Wnt (19). Dkk2 seems able to act as agonist as well as antagonist for Wnt/LRP6 signaling depending on the cellular context and therefore co-factors such as krm2 (18–20). In EOC Zhu et al. suggest that Dkk2 may functions as a Wnt pathway inhibitor (13).

Estrogen (E2, 17β-estradiol) has numerous cellular functions in the human body including gynecologic cancer biology (21) and interactions between estrogen and Wnt signaling have been described (22–25). In this context an interplay of Dkk2 and estrogen receptors (ER) could link these two mechanisms and classical nuclear ERα or ERβ as well as the G protein-coupled estrogen receptor (GPER) could be involved in this process.

GPER is a transmembrane receptor with intracellular domains binding E2 (26), which mediates rapid non-genomic estrogen signaling (27). Its activation *via* agonists like G1 or E2 (28) leads to cAMP production (29), activation of extracellular signal-related kinase 1 and 2 (Erk1/2) (28), mobilization of intracellular Ca^2+^, phosphatidylinositol 3-kinase (PI3K) activation (26) and the induction of metalloproteinases which then transactivates the epidermal growth factor receptor (30). GPER can also indirectly impact gene transcription (31). Since its role in ovarian cancer has been conflicting so far (32–34) this analysis focused on the correlation of Dkk2 with GPER to identify a possible link between Wnt and estrogen and investigating their potential prognostic significance.

## Methods

### Patients

In this study 156 formalin-fixated and paraffin-embedded tissue specimens of epithelial ovarian cancer from patients who had been treated in the Department of Obstetrics and Gynecology at Ludwig-Maximilians-University of Munich between 1990 and 2002 were analyzed. Numerous markers were already examined in this collective in preceding studies (35–37). Clinical data was collected from the patient’s charts and information about the follow up was acquired from the Munich Cancer Registry.

Only patients with malignant, non-borderline tumors were included in the study. Seventy-three patients (46.8%) were older or age 60 years at the initial diagnosis and 83 patients (53.2%) were younger than 60 years. There were no data available about estrogen replacement therapy in postmenopausal women. Pathologists categorized the histological subtypes of the samples: LGSC (n=24), HGSC (n=80), endometrioid (n=21), clear cell (n=12), mucinous (n=13). According to the updated FIGO classification from 2014, specimens of serous ovarian cancer were re-evaluated and attributed to low-grade (G1) and high-grade (G3) histology. Endometrioid and mucinous ovarian cancer samples were related to G1, G2, and G3. Clear cell cancer was always categorized as G3 (38). Staging was done following the FIGO classification: I (n=35), II (n=10), III (n=103), IV (n=3) (**Table 1**).

**Table 1:** Correlation between Dkk2 expression and clinicopathologic characteristics of ovarian cancer patients.

Characteristics		Total	Dkk2 low expression	Dkk2 high expression	P value
		Number of cases(%)	Number of cases	Number of cases	
Age(y)					
	≥60y	73 (46.8)	28	21	**<0.001**
	<60y	83 (53.2)	17	38	
Tumor histology					
	LGSC	24 (15.4)	2	17	**0.001**
	HGSC	80 (51.3)	28	27	
	Clear cell	12 (7.7)	0	6	
	Endometrioid	21 (13.5)	7	4	
	Mucinous	13 (8.3)	6	2	
	Missing	6 (3.8)			
FIGO					
	I	35 (22.4)	8	14	0.615
	II	10 (6,4)	2	4	
	III	103 (66.0)	33	39	
	IV	3 (1.9)	1	0	
	Missing	5 (3.2)			
Expression of GPER					
low expression (IRS ≤ 8)		83 (53.2)	33	28	**0.005**
high expression (IRS>8)		70 (44.9)	11	31	
				

Dkk2, Dickkopf2; GPER, G protein-coupled estrogen receptor; HGSC, high-grade serous carcinoma; LGSC, low-grade serous carcinoma; FIGO, International Federation of Gynecology and Obstetrics. Bold numbers represent p-values < 0.05.

### Sampling and Microarray Construction

Three core biopsies for each EOC patient were taken from paraffin-embedded and formalin-fixed tumor blocks in our archive. The biopsies were assembled in tissue microarrays (TMA) paraffin blocks. Those TMA paraffin blocks were cut into serial sections at 2 μm and fixed on slides. A pathologist verified that representative areas of the tumor were aligned on the slides.

### Immunohistochemistry

Immunohistochemical staining of paraffin-embedded and formalin-fixed tissue micro arrays of ovarian cancer specimens for Dkk2 was performed as previously described (39). The TMA slides were dewaxed in Roticlear (Carl Roth Karlsruhe, Germany) for 20 min. The endogenous peroxidase was suppressed with 3% hydrogen peroxide (Merck, Darmstadt, Germany) in methanol (20 min). The specimens were rehydrated in a descending alcohol series (100%, 70%, 50% ethanol). The epitopes were retrieved by putting the slides in a pressure cooker with sodium citrate buffer (pH 6.0) for 5 min. After cooling to room temperature, the slides were washed in in distilled water and phosphate-buffered saline (PBS). To evade unspecific staining reagent 1 of the polymer kit (ZytoChem Plus HRP Polymer System, Berlin, Germany) was administered for 5 min. Next the slides incubated at +4°C for 16 h with the primary anti-body Anti-Dkk2 polyclonal rabbit IgG (ProteinTech, Manchester, UK). As negative controls the primary antibody was replaced by normal rabbit immunoglobulin G([IgG] supersensitive rabbit negative control; BioGenex, Fremont, California). Washing in PBS and the application of reagents 2 (20 min) and 3 (30 min) of the polymer kit anticipated the substrate-staining with chromogen diaminobenzidine (Dako, Hamburg, Germany). Counterstaining in Mayer acidic hematoxylin (Waldeck-Chroma, Münster, Germany) and dehydration in an ascending series of alcohol followed by Roticlear was performed. Cervical tissue was served as positive control.

Using a microscope (Leitz, Wetzlar, Germany) the immune-reactivity score (IRS) was applied to assess the immunostaining extent semi-quantitatively by two blinded examiners. The IRS is composed of the staining intensity (0=negative, 1=low, 2=moderate, 3=strong) multiplied with the percentage of stained cells (0=no staining, 1%≤10% positive cells, 2 = 11%–50% positive cells, 3 = 51%–80% positive cells, 4%≥81% positive cells). The immunoreactivity score ranges from 0 to 2: negative, 3 to 4: weak, 6 to 8: moderate, and 9 to 12: strong (40). Formerly published staining results of GPER in this panel recorded in the archive of the laboratory were recaptured (36).

### Staining Evaluation

In order to define reliable cut-off points for the IRS of the Dkk2 staining the receiver operating characteristics (ROC) curve was used. ROC curve illustrates sensitivity on the y-axis plotted against (1-specificity) on x-axis (41). With Youden Index (42) the optimal cut-off was defined with highest possible values for sensitivity and specificity. For the Dkk2 staining IRS 0-4 was considered as weak and IRS 6-12 as high. Regarding its components, the IRS can never have a value of 5. GPER expression was divided into low (IRS ≤ 8) vs. high (IRS>8) according to the median (36).

### Statistical Analysis

Statistical analysis was operated with SPSS 25 (IBM, Chicago, IL, USA). With the Kruskal-Wallis analysis the null hypothesis was tested against its opposite. Further Spearman’s correlation analysis and Kruskal-Wallis analysis was applied for testing correlation of Dkk2 and GPER scores. The Kaplan-Meier estimate was used for analyzing times to event variables. Correlations between mean Dkk2 expression and clinicopathologic characteristics were assessed with Chi-Square tests (**Table 1**, crosstab). For all tests p-values ≤ 0.05 were considered as statistically significant. Figures were designed with SPSS 25 and Microsoft Power Point 2016 (Microsoft, Redmond, WA, USA).

## Results

Correlations between Dkk2 expression and clinicopathologic characteristics of EOC patients are displayed in **Table 1**. A median IRS of 6 for anti-Dkk2 staining was observed in the 131 of 152 cases (86%) with adequate staining. Applying ROC curve analysis, an IRS>4 was selected as cut-off.

**Figure 1:**
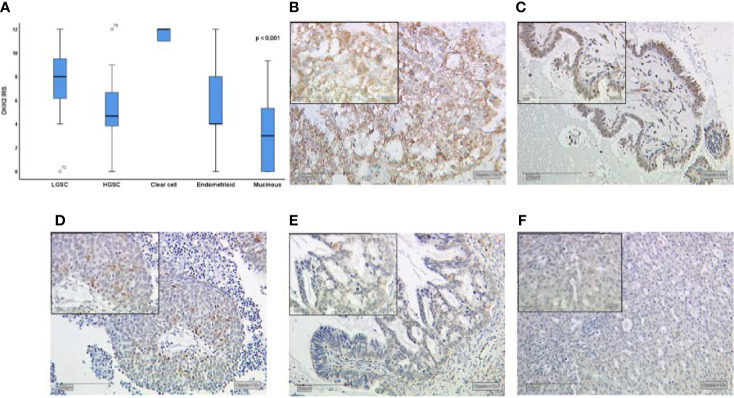
Dkk2 expression patterns in different histological subtypes of EOC after immunohistochemical staining was performed as shown in a Kruskal-Wallis analysis for histological subtypes **(A)**. Clear cell carcinomas **(B)** presented the strongest staining patterns. Low-grade serous carcinomas (LGSC; **C**) had shown moderate Dkk2 expression. For endometrioid **(D)**, mucinous **(E)** and high-grade serous carcinomas (HGSC; **F**) the median IRS was lower. Scale bares equal 200 μm.

Dkk2 expression differed significantly between the histological cancer subtypes (**Figures 1A–F**) with clear cell carcinomas showing the highest median IRS of 12 compared to the other subtypes (range: 9–12; p<0.001). While endometrioid and mucinous EOCs exhibited both a median IRS of 4, the overall cohort of serous EOCs had moderate staining extent at IRS of 6 which subdivided into high-grade serous histology with an IRS of 4 (range 0–12) and significantly higher for low-grade serous histology with an IRS of 6 (range 4–12; p<0.001).

**Figure 2:**
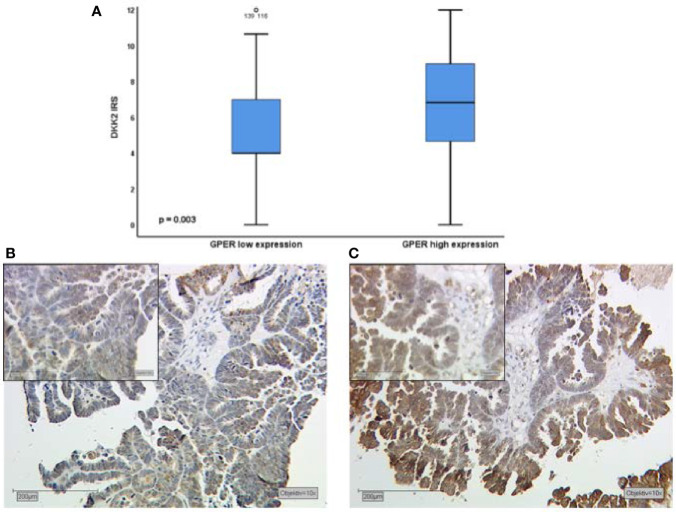
Kruskal-Wallis analysis for correlation of Dkk2 and GPER expression **(A)**. High expression of Dkk2 **(B)** correlates with high GPER expression **(C)** in tissue samples of the same patient. Scale bares equal 200 μm.

Performing correlation analysis of Dkk2 expression and clinicopathological parameters such as distant metastasis, affected lymph nodes, FIGO classification, and grading, no significant results were found. In addition, Dkk2 expression was examined in comparison to other potentially pathological markers with a possible impact on the prognosis of EOC. Cytoplasmatic Dkk2 was observed to correlate significantly with cytoplasmic GPER expression (cc=0.304, p=0.001). Further analysis revealed that high Dkk2 expression is correlated to high GPER expression (**Figure 2**). In contrast, Dkk2 did not correlate with either ERα or ERβ expression (**Table 2**).

**Table 2:** Results of Spearman’s correlation analysis of Dkk2 with the different estrogen receptors (GPER, ERα, ERβ).

Staining	DKK2	GPER	*ER*α	ERβ
**DKK2**				
cc	1.000	**0.304**	0.092	-0.080
p	.	**0.001**	0.298	0.366
n	125	124	131	128

Dkk2, Dickkopf 2; GPER, G protein-coupled estrogen receptor; cc, correlation coefficient; p, two-tailed significance; n, number of patients. Bold numbers represent p-values < 0.05.

**Figure 3:**
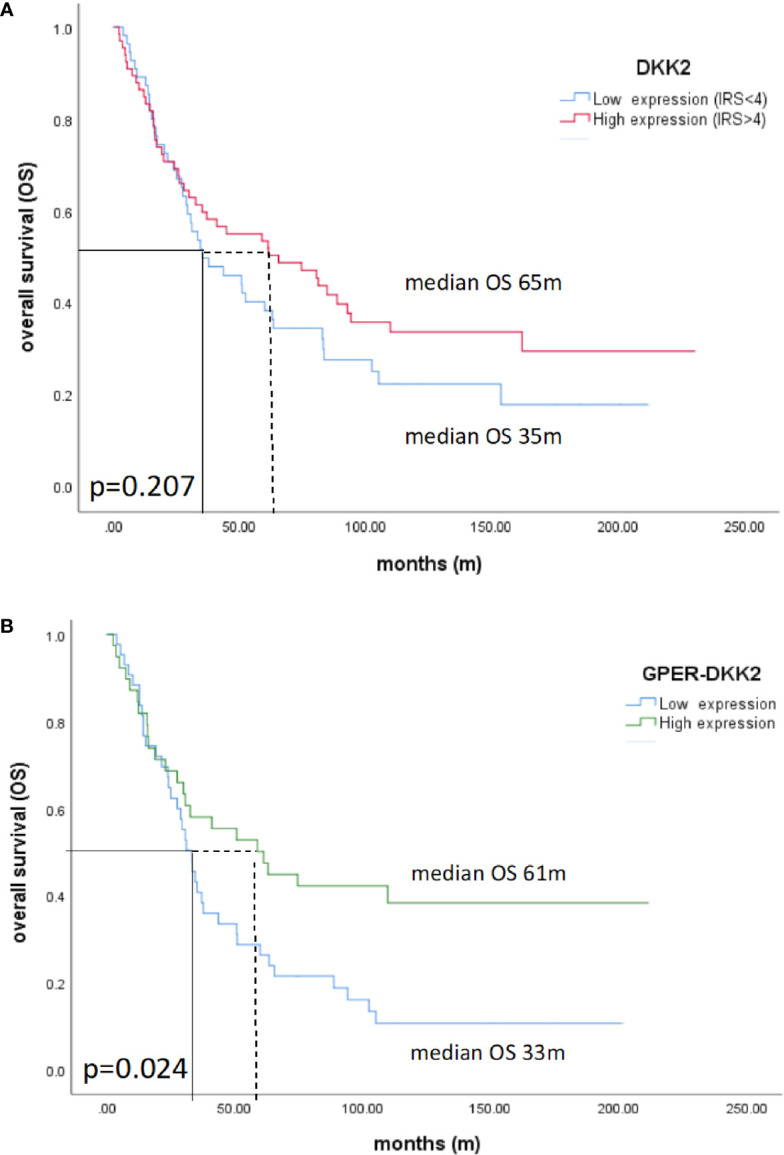
Kaplan-Meier estimates of Dkk2 **(A)** and Dkk2 combined with GPER expression **(B)** were analyzed. Though not statistically significant, high cytoplasmic Dkk2 **(A)** and GPER (36) expression was connoted with a longer OS. Patients with carcinomas highly expressing both Dkk2 and GPER in the cytoplasm compared to patients with carcinomas lowly expressing Dkk2 and GPER showed significantly (61 months vs. 33 months, p=0.024) increased OS **(B)**.

Patients with high Dkk2 expression (IRS>4) exhibited longer OS with a median of 65 months compared to 35 months in Kaplan-Meier analysis, although this difference was not statistically significant (p=0.207; **Figure 3A**). The same trend was observed for GPER expression as published before with longer OS for patients with high expression but without statistical significance (36). When the expression analyses of the two markers were combined, patients with high Dkk2 (IRS>4) as well as high GPER (IRS>8) expression had a significantly longer OS with 61 months compared to 33 months in patients with low expression of both influenced OS (p=0.024; **Figure 3B**).

## Discussion

Dkk2 as a Wnt/β-catenin antagonist may play an important role in ovarian cancer (13, 18, 43). In this analysis, we investigated the expression of Dkk2 in the different histological subtypes of epithelial ovarian cancer, its relation to clinicopathological aspects and its impact on OS. Clear cell carcinoma exhibited the highest Dkk2 expression at all and LGSC showed significantly higher expression compared to the other histologies, which could reflect the different pathogenesis and origins of the histological subtypes (44).

In a previous study from Zhu et al. it has been shown that Dkk2 is frequently methylated and therefore epigenetically silenced in ovarian cancer. Lower Dkk2 expression levels correlated with tumor progression and advanced tumor stages (FIGO III-IV). By treating mice with the DNA methyltransferase inhibitor 5- aza-2′-deoxycitidine (decitabine) in order to re-establish Dkk2 expression in mice tumor growth was impaired (13). This is in accordance with our findings, suggesting an impact of Dkk2 on OS although this was not significant. Seemingly aberrant DNA methylation patterns also play a major role in platinum resistance, therefore the potential of epigenetic modulator decitabine to restore sensitivity towards platinum has been successfully tested in a phase II clinical trial (45). So far, agents for epigenetic therapy may cause severe adverse effects, in particular when they are administered in combination with chemotherapy. This underscores the necessity of more selective epigenetic modulators (46).

The impact of GPER on the OS of ovarian cancer patients has been controversially discussed so far (32–34). The conflicting results in these studies may arise from application of different concentrations for the agonists E2 and G1 and the investigation in different cancer cell lines. Accounting for these and the current results, GPER may not be sufficient to predict OS on its own. However, in combination with other factors like Luteinizing Hormone/Choriogonadotropin Receptor and Follicle Stimulating Hormone Receptor (36) or Dkk2, it could serve as a positive prognostic factor for patients suffering from epithelial ovarian cancer.

As previous studies elucidated a possible connection between estrogen and Wnt signaling (22–25), we investigated the relationship of Dkk2 with estrogen receptors. Subcellular localization of the DKK2 staining pattern was noted which has been previously attributed to the Golgi apparatus (www.proteinatlas.org). Unlike other studies in breast cancer which have shown an association between plasma membrane expression and outcome, plasma membrane expression of GPER was not detected in the ovarian cancer samples evaluated here (47). We could demonstrate a strong correlation of high cytoplasmic Dkk2 and high cytoplasmic GPER expression levels in EOC samples. In contrast, no correlation of Dkk2 with the traditional estrogen receptors ERα or ERβ was noted. To the best of our knowledge, a possible connection of GPER and Dkk2 has not yet been investigated. The described association of higher Dkk2 expression in younger patients may be reflected by more patients in premenopausal status and therefore relate to the estrogen levels in these patients.

In our study, a high Dkk2 expression in combination with a high cytoplasmic level of GPER had a significant prognostic impact on OS which might help to find new approaches for possible treatment strategies accounting for the correlation of estrogen and Wnt signaling pathways. As Dkk2 is a modulator of the Wnt pathway, therapeutics addressing this cascade could be combined with agents modulating GPER. Although promising in early stage development, previous strategies targeting Wnt proteins like tumor associated MUC1 (TA-MUC1) inhibitor gatipotuzumab and others have not led to durable responses and not reached clinical significance so far (48). Very recently, a Wnt modulator of Dkk1 (DKN-01) has shown interesting activity and is currently in a phase 2 basket trial which still supports the rationale for this approach (49).

In renal cancer cells, the selective estrogen receptor modulator genistein reportedly abolished miR-1260b, which is able to suppress Wnt signaling modulators like Dkk2, and therefore preserved levels of these proteins (24). Genistein is not exclusively binding to GPER though, it also inflects ERα and/or ERβ (50). In hepatocytes administering the GPER antagonist G15 attenuated β-cat Ser675 phosphorylation and T-cell factor (TCF) expression suggesting an involvement of GPER in β-cat/TCF activities (51). Beside cell culture experiments, analyzing methylation patterns with methylation-specific polymerase chain reaction could help to further investigate the suggested interactions of GPER and Dkk2. Implementing TCF/LEF (lymphoid enhancing factor) reporter assays, could be assessed to evaluate possible effects of GPER agonists or antagonists on the Wnt signaling pathway.

There are some factors limiting our study. First of all, it is retrospective based on a single dataset with a relatively low sample size which may not be sufficient to elucidate all subtype-specific differences in an heterogenous tumor like ovarian cancer (44). Additional specific information of patient characteristics like an history of hormonal replacement therapy could enrich the investigation how estrogen levels interact with Dkk2 and better account for possible environmental toxicants. In Kaplan-Meier analysis, subtype-specific evaluation did not reveal significant differences regarding OS between patients with high and low Dkk2 expression so that results can be considered as a base for further research in ovarian cancer. Further methods will be necessary capture the extensive complexity of GPER and Wnt signaling pathways with their possible interaction as indicated.

However, aside from these limitations our data is in accordance with previous findings in EOC literature (13, 33, 36, 45, 52) and elucidate that targeting the GPER receptor as well as the Wnt pathway could represent promising therapeutic strategy in ovarian cancer. The study might provide an impetus to further investigate the crosstalk between estrogen and Wnt signaling in regard to the therapeutic potential in EOC.

## Data Availability Statement

The raw data supporting the conclusions of this article will be made available by the authors, without undue reservation.

## Ethics Statement

The studies involving human participants were reviewed and approved by Ludwig-Maximilians-University, Munich, Germany. Written informed consent for participation was not required for this study in accordance with the national legislation and the institutional requirements.

## Author Contributions

PF, UJ, SH, AB, SM, and FT contributed conception and design of the study. DM performed histological examinations on the patient tumour tissue. PF, CK, UJ, and PR did the laboratory work. PF, TK, UJ, and PR performed the statistical analysis. PF and UJ wrote the first draft of the manuscript. All authors contributed to the article and approved the submitted version.

## Funding

This study was funded by the Medical Faculty of the LMU Munich.

## Conflict of Interest

SM received research support, advisory board, honoraria and travel expenses from AstraZeneca, Clovis, Medac, MSD, PharmaMar, Roche, Sensor Kinesis, Tesaro, and Teva. FT declares research support, advisory board, honoraria and travel expenses from AstraZeneca, Medac, PharmaMar, Roche, and Tesaro. SH reports grants from Baden-Württemberg Ministry of Science, Research and the Arts, from StuRa Ruprecht-Karls-University of Heidelberg, FöFoLe LMU Munich Medical Faculty, grants from FERRING, personal fees from Roche, other from Astra Zeneca, grants from Novartis Oncology, grants and non-financial support from Apceth GmbH, non-financial support from Addex and grants from Heuer Stiftung. She further reports grants from Deutsche Forschungsgemeinschaft within the funding program Open Access Publishing, by the Baden-Württemberg Ministry of Science, Research and the Arts and by Ruprecht-Karls-University Heidelberg, outside the submitted work. TK receives a grant from the Friedrich-Baur-Stiftung.

The remaining authors declare that the research was conducted in the absence of any commercial or financial relationships that could be construed as a potential conflict of interest.

## Abbreviations

Dkk2, Dickkopf2; EOC, epithelial ovarian cancer; E2, estrogen, 17β-estradiol; ERα, nuclear estrogen receptor alpha; ERβ, nuclear estrogen receptor alpha beta; Erk1, extracellular signal-related kinase 1; Erk2, extracellular signal-related kinase 2; GPER, G protein-coupled estrogen receptor; HGSC, high-grade serous carcinoma; IRS, immune-reactivity score; LGSC, low-grade serous carcinoma; LRP5, low-density-lipoprotein-related protein 5; LRP6, low-density-lipoprotein-related protein 6; OS, overall survival; PI3K, phosphatidylinositol 3-kinase; ROC curve, receiver operating characteristics curve; TCF, T-cell factor; TMA, tissue microarrays.
